# Neoehrlichiosis Causing Hemolytic Anemia in Patients Treated with Rituximab, Norway, 2024–2025

**DOI:** 10.3201/eid3209.260553

**Published:** 2026-09

**Authors:** Martin Paulson, Christine Wennerås, Hanne Quarsten, Kristine K. Berg, Anders L. Drivenes, Magnus Moksnes, Tor Henrik A. Tvedt

**Affiliations:** Vestfold Hospital Trust, Tønsberg, Norway (M. Paulson, M. Moksnes); University of Gothenburg, Gothenburg Sahlgrenska Academy, Gothenburg, Sweden (C. Wennerås); Sahlgrenska University Hospital, Gothenburg (C. Wennerås); Sørlandet Hospital Trust, Kristiansand, Norway (H. Quarsten, K.K. Berg); Oslo University Hospital, Oslo, Norway (A.L. Drivenes, T.H.A. Tvedt); University of Oslo Institute of Clinical Medicine, Oslo (T.H.A. Tvedt)

**Keywords:** neoehrlichiosis, Anaplasmataceae, Neoehrlichia mikurensis, tickborne diseases, bacteria, bacterial infections, vector-borne infections, rituximab, immunocompromised patients, hemolytic anemia, splenomegaly, Norway

## Abstract

Neoehrlichiosis is caused by infection with the tickborne intracellular bacterium *Neoehrlichia mikurensis*. We describe 3 cases of direct antiglobulin test–negative hemolytic anemia in rituximab-treated patients infected with tickborne *N. mikurensis* bacteria in Norway during 2024–2025. All 3 patients had splenomegaly and night sweats. The hemolysis ranged from mild with compensated hemolysis to severe transfusion-dependent hemolysis that resulted in splenectomy in 1 case. All patients experienced complete resolution of hemolytic anemia and other symptoms of infection after eradication therapy with doxycycline for 21 days. Neoehrlichiosis should be considered as a curable cause of hemolytic anemia in *N*. *mikurensis*–endemic regions.

The tickborne intracellular bacterium *Neoehrlichia mikurensis* is the cause of the infectious disease neoehrlichiosis (*1*). The initial case reports of neoehrlichiosis were published from various countries in Europe in 2010 (*2*–*4*). The typical manifestations of neoehrlichiosis includes signs and symptoms of systemic inflammation, such as fever, malaise, fatigue, and nightly sweats, as well as myalgia, skin rashes and vascular events (e.g., venous thromboembolism and vasculitis) (*1*,*5*,*6*). Vascular manifestations probably are attributable to *N*. *mikurensis* being an intracellular pathogen that can grow and propagate in vascular endothelium (*7*). The infection has a variable clinical manifestation, ranging from asymptomatic carriage to life-threatening disease (*3*,*4*,*8*,*9*). The heterogenous clinical features of the disease, including manifestations such as vasculitis resembling giant cell arteritis and polyarteritis nodosa and life-threatening hemophagocytic lymphohistiocytosis, illustrate the complexity of neoehrlichiosis (*6*,*9*).

Compromised B-cell immunity and splenectomy are risk factors for neoehrlichiosis (*5*). Of the published cases of neoehrlichiosis, most were in patients who were immunocompromised and had frequently been treated with the B-cell lymphocyte–depleting agent rituximab (*9*–*12*). Secondary anemia is a frequent finding in patients with neoehrlichiosis that could be caused by long duration of the infection attributable to diagnostic delay (*1*,*9*,*13*). Neoehrlichiosis is not detected by blood culture and currently only can be diagnosed by molecular detection of bacterial DNA. One study of 103 patients with neoehrlichiosis in Sweden found a 3% prevalence of autoimmune hemolytic anemia (*5*). In addition, a case report describes Coombs test-positive hemolytic anemia, neutropenia, and thrombocytopenia in a dog infected with *N*. *mikurensis* (*14*). We describe 3 patients from southeastern Norway who had been treated with rituximab and who had splenomegaly, direct antiglobulin test (DAT)–negative hemolytic anemia, and infection with *N*. *mikurensis*. 

## Methods and Patients

All 3 patients were treated at Vestfold Hospital Trust, Tønsberg, Norway. Patient 1 also underwent diagnostic workup and treatment at Oslo University Hospital’s Department of Hematology (Oslo, Norway). We obtained written informed consent from all patients for the publication of their case reports.

We performed the polyspecific DAT tests for IgG and C3d at Vestfold Hospital Trust. We performed DAT tests for IgG, C3d, IgM, and IgA at Oslo University Hospital, Rikshospitalet (Oslo).

### PCR Assays

We tested DNA isolated from the plasma or buffy coat fractions of whole blood by using real-time reverse transcription PCR for the detection of *Borrelia burgdorferi* (*ospA* and 16S rRNA genes), *Borrelia miyamotoi* (16S rRNA gene), *Anaplasma phagocytophilum* (*groEL* gene), *Rickettsia* spp. (*gltA* gene), and *N*. *mikurensis* (*groEL* gene, named CNM-II in the reference article) at the Department of Medical Microbiology at Sørlandet Hospital (Kristiansand, Norway) using primers and probes as described previously (*8*). We performed *Babesia* subspecies DNA PCR at Statens Serum Institut (Copenhagen, Denmark). We established a diagnosis of hemolysis on the basis of reduced hemoglobin, reduced plasma haptoglobin, increased plasma lactate dehydrogenase, and increased blood reticulocytes.

## Case Reports

### Patient 1

A 61-year-old woman sought care for rapid-onset dyspnea, fatigue, palpitations, dizziness, and a 6-month history of infrequent febrile episodes. Her medical history included rheumatoid arthritis for 17 years, breast cancer treated 16 years previously, and episodic severe depression for 1.5 years. Her arthritis initially was treated with oral methotrexate, salazopyrine, and hydroxychloroquine. Methotrexate temporarily was withdrawn because of cytopenias after a year and permanently withdrawn when she had breast cancer diagnosed. Her breast cancer was treated with surgery after 6 courses of neoadjuvant chemotherapy with fluorouracil, epirubicin hydrochloride, and cyclophosphamide, followed by postoperative radiotherapy. After completion of breast cancer treatment, treatment for rheumatoid arthritis commenced with rituximab infusions (1,000 mg 2×/y). She also was treated with venlafaxine for depression and anxiety.

At admission, we diagnosed severe transfusion-dependent direct antiglobulin test (DAT)–negative hemolytic anemia in the patient (Table). We suspected drug-induced hemolytic anemia and discontinued venlafaxine and rituximab, which later were reinstated because of lack of improvement. Two months after initially seeking care, the patient was admitted to a hospital with acute appendicitis and worsened hemolysis. After appendectomy, we initiated prednisolone (1 mg/kg/d), which resulted in partial reduction of hemolysis. We introduced the interleukin 1 receptor inhibitor anakinra 7 months later as a steroid-sparing agent after relapses of fever and worsened hemolysis during the tapering of prednisolone.

**Table:** Characteristics, reported symptoms, and biochemical test results at admission, during disease course, and post-treatment with antibiotics for 3 patients with neoehrlichiosis, hemolytic anemia, and splenomegaly, Norway, 2024–2025*

Characteristic	Patient 1	Patient 2	Patient 3
Age, y/sex	61/F	62/M	48/M
Indication for rituximab	Rheumatoid arthritis	Myasthenia gravis	Multiple sclerosis
Immunosuppression	Rituximab 1,000 mg 2×/y	Rituximab 1,000 mg 2×/y	Rituximab, 1,000 mg 1×/y
Signs and symptoms			
Fever	Yes	No	Yes
Night sweats	Yes	Yes	Yes
Weight loss	Yes	No	Yes
Fatigue	Yes	No	Yes
Skin involvement	No	Hyperpigmented rash of the lower extremities	No
Vascular involvement	Yes	No	No
Hemolysis	Yes	Yes	Yes
Direct antiglobulin test	Negative	Negative	Negative
Blood biochemistry at admission/peak or nadir during disease course/after treatment (reference range)			
Blood Hb, g/dL	6.4/5.8/12.1 (11.7–15.3)	9.6/8.8/14.7 (13.4–17.0)	13.8/17.5/17.5 (13.4–17.0)
Blood platelets × 10^9^/L (145–390)	416/932/373	234/154/314	186/241/241
Blood leukocytes × 10^9^ cells/L (3.5–10.0)	4.75/2.74/6.0	4.93/3.32/5.97	5.32/5.72/4.19
Blood reticulocytes × 10^9^ cells /L (30–100)	336/70/120	180/48/78	103/52/84
Plasma C-reactive protein, mg/L (<5)	15/92/<1.0	3.1/3.4/<1.0	11/1.7/<1.0
Plasma ferritin, µg/L (30–400)	950/8,004/3,274	92/41/106	362/NA/NA
Plasma haptoglobin, g/L (0.50–2.10)	0.34/<0.10/0.45	<0.1/<0.1/0.24	<0.1/<0.1/0.54
Plasma, LDH, U/L (35–210)	189/261/138	286/159/188	288/164/173
Plasma, bilirubin (total) µmol/L (<25)	77/100/20	19/10/12	19/22/16
Blood type	A RhD+	NA	NA
Plasma IgG, g/L (6.10–14.9)	7.29/6.91/8.68	10.4/NA/NA	8.63/8.63/9.42
*Neoehrlichia* *mikurensis* PCR Ct value (at admission)	22	28	25
Largest spleen size before treatment (radiologic imaging)	13.8-cm coronal length	17-cm coronal length	17.7-cm coronal length, hilar width 7.4 cm
Treatment and results			
Time from treatment start to improved hemolysis	30 d to normalization of reticulocytosis and Hb increase >2 g/dL	60 d to normalization of reticulocytosis and Hb increase >2g/dL	47 d to normalization of reticulocytosis and Hb increase >2 g/dL
Time from treatment to improved symptoms	Cessation of fever after 3 d	NA	Cessation of night sweats and fatigue after 7 d
Spleen size posttreatment (radiologic imaging)	Splenectomized	Not evaluated	14 cm coronal length, hilar width 5.2 cm

*Ct, cycle threshold; Hb, hemoglobin; LDH, lactate dehydrogenase; NA, not available.

Diagnostic tests of blood and bone marrow revealed no evidence of congenital or acquired erythrocyte membrane defects, including paroxysmal nocturnal hemoglobinuria (PNH), lymphoproliferative disorders, or other hematologic neoplasia. Results of a 184-gene panel of hereditary causes of anemia and bone-marrow failure (e.g., Fanconi anemia, Diamond-Blackfan anemia, and hereditary stomatocytosis) were negative. No findings were suggestive of Wilson disease (i.e., normal ceruloplasmin and copper serum and urine levels) or of zinc- or arsenic-related poisoning. Computed tomography (CT) scans of the neck to pelvis were unremarkable, whereas a fluorodeoxyglucose (FDG) positron emission tomography (PET)–CT scan showed a slight uptake in the spleen initially deemed to be reactive. When a repeat FDG PET–CT scan 3 months later showed a maintained slight uptake in the spleen, the patient underwent diagnostic splenectomy because of a continued suspicion of underlying lymphoproliferative disorder. Before splenectomy, the patient had thrombophlebitis in the right cubital fossa and right ankle diagnosed. After splenectomy, hemoglobin levels improved for a period, but evidence of hemolysis remained. Histopathologic examination of the spleen showed depletion of B cells in the white pulp, secondary to rituximab treatment, and expanded red pulp with extramedullary hematopoiesis but no evidence of lymphoma. Within 2 months, the patient’s hemolytic anemia relapsed. Because of a history of tick exposure and erythema migrans a few years prior, we sent EDTA blood samples for PCR testing for tickborne microbes, including *Rickettsia* spp., *B*. *miyamotoi*, *B*. *burgdorferi*, *A*. *phagocytophilum, N*. *mikurensis* (*8*), and *Babesia* subsp. The patient tested positive for *N*. *mikurensis*, upon which we initiated oral doxycycline (100 mg 2×/d for 21 d). Her symptoms, most prominently fever, night sweats and fatigue, as well as all signs of hemolysis, rapidly improved and cleared completely (Table; Figure). She tested negative for *N*. *mikurensis* DNA by PCR after the course of antibiotics. We also detected *N*. *mikurensis* DNA in historical serum samples drawn 22, 11, and 6 months before the time of diagnosis. At a 12-month follow-up, no evidence of hemolytic anemia remained.

**Figure:**
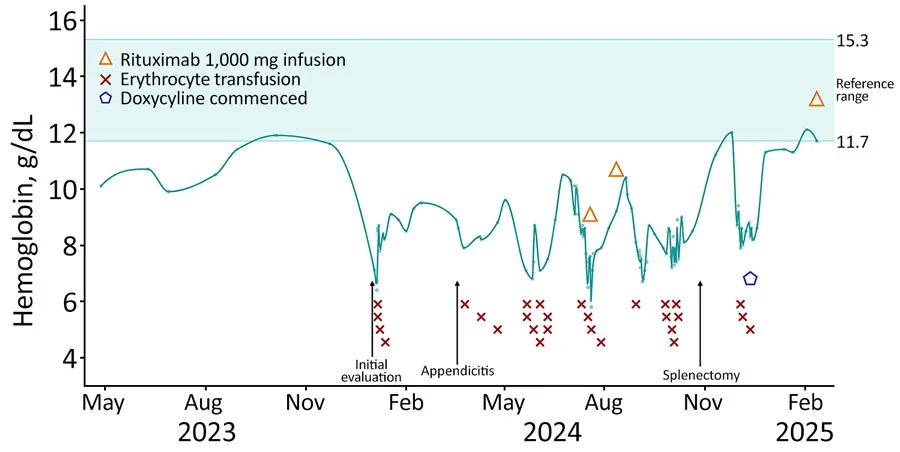
Hemoglobin levels (blue line) for patient 1 in a case series of 3 patients with neoehrlichiosis and hemolytic anemia at admission, during disease course, and after treatment with antibiotics, Norway, 2024–2025.

### Patient 2

A 62-year-old man was referred to our outpatient clinic with a 4-month history of gradually progressive symptomatic anemia, prominent fatigue, and night sweats. He did not have fever, weight loss, nor any current or previous vascular events. His medical history included myasthenia gravis for 14 years that had been treated with rituximab infusions (1,000 mg 2×/y) for the previous 5 years. One year earlier, he had a prolonged SARS-COV-2 infection, evaluated with a chest CT, with an incidental finding of a slightly enlarged spleen. Laboratory findings at referral showed mild DAT-negative hemolytic anemia (Table). A peripheral blood smear showed no evidence of cellular dysplasia, blasts, or schistocytes. A test result for PNH was negative. We performed coagulation tests because of easy bruising, but we detected no coagulation defect. The patient had had no recall of tick bites. We performed no testing for SARS-COV-2. Because of several similarities to the first patient case (e.g., a probable nonimmunologic hemolytic anemia, treatment with rituximab, and slight splenomegaly), we performed PCR testing for tickborne microbes, including *Rickettsia* spp., *B*. *miyamotoi*, *B*. *burgdorferi*, *A*. *phagocytophilum, N*. *mikurensis*, and *Babesia* subsp. The patient was positive by PCR for *N*. *mikurensis*, and we initiated oral doxycycline (100 mg 2×/d for 21 d). A month later, his anemia had improved, although haptoglobin still was suppressed slightly, indicating low-grade hemolysis. Two months after the patient finished doxycycline treatment, hemoglobin and haptoglobin levels were normal (Table). At 12-month follow-up, no evidence of hemolytic anemia remained.

### Patient 3

A 48-year-old man was referred our care with splenomegaly and profuse night sweats that had started 1 year earlier. His medical history included relapsing or remitting multiple sclerosis 5 years prior, which had been treated with rituximab infusions (1,000 mg 1×/y) for the previous 2 years. CT imaging of the thorax, abdomen, and pelvis done a few months before referral revealed no pathologic lymphadenopathy but substantial splenomegaly (largest diameter 17.7 cm). One month before his first episode of night sweats, he had traveled to the southern United States, Caribbean, and South America, none of which warranted malarial chemoprophylaxis. He had not contracted fever, insect bites, or rashes during his travels. He had a history of tick bites after hiking trips in southeastern Norway but no erythema migrans. Blood chemistry taken a week before the appointment at our facility showed mild and fully compensated DAT-negative hemolytic anemia (Table). The levels of haptoglobin, reticulocytes, and lactate dehydrogenase normalized spontaneously 10 days later.

An ultrasound at the time of the first consultation showed a reduction of spleen size to 15.1 cm (largest diameter). PNH test result was negative, and peripheral blood smears results were normal. Bone marrow trephine biopsy showed a very slight increase of intrasinusoidal T-lymphocytes of uncertain importance. Repeated bone marrow trephine biopsies and bone marrow aspirate analyses by flow cytometry yielded similar results. A FDG PET or CT scan showed no pathologic FDG uptake in the liver, spleen, or bone marrow and a maximal spleen diameter of 15.8 cm. We noted only a slight clonal T-cell receptor rearrangement in the bone marrow and normal results in peripheral blood, consistent with a reactive cause with no conclusive evidence of lymphoproliferative disorder. A repeat ultrasound of the spleen a few months later showed a spleen size of 14.3 cm. Six months after the initial diagnostic work-up and 2 months after receiving his yearly rituximab infusion, the patient experienced a relapse of symptoms with increasing night sweats, increase in spleen size to 17.4 cm on ultrasound, and hemolysis with a substantial reduction in hemoglobin levels and signs of intravascular hemolysis (Table). 

We performed testing for tickborne microbes, including *Rickettsia* spp., *B*. *miyamotoi*, *B*. *burgdorferi*, *A*. *phagocytophilum* and *N*. *mikurensis*, and the patient was positive by PCR for *N*. *mikurensis*. We did not test for *Babesia* subsp. We initiated treatment of the patient with oral doxycycline (100 mg 2×/d for 21 d). A repeat ultrasound of the spleen performed 6 weeks later showed a reduction in size to 14 cm. His symptoms improved and resolved within a week of antibiotic treatment. A PCR test for *N*. *mikurensis* was negative after treatment. We also detected *N*. *mikurensis* DNA in stored serum samples drawn 12 and 8 months before the time of diagnosis. At 12-month follow-up, no evidence of hemolytic anemia remained.

## Discussion

We describe 3 patients with a diagnosis of DAT-negative hemolysis, splenomegaly, and infection with *N*. *mikurensis*. We considered neoehrlichiosis to be the cause of their hemolysis, and all patients went into remission after treatment with doxycycline. Receipt of rituximab is a risk factor for neoehrlichiosis, and splenomegaly and systemic symptoms are established features of neoehrlichiosis. Two of the 3 patients had documented *N*. *mikurensis* DNA in archival serum samples up to 2 years before the time of diagnosis. Although no archived blood samples from the second patient were available, he had splenomegaly of unknown cause 1 year previously, which in retrospect might have been caused by *N*. *mikurensis*. Neoehrlichiosis may progress from asymptomatic latent infection to symptomatic acute infection. A recent study showed that *N*. *mikurensis* causes latent infections that can reactivate when B-cell immunity is suppressed by rituximab (*15*). The mechanism by which *N*. *mikurensis* causes hemolysis is unclear but does not seem to involve either complement antibodies or IgG. 

Hemolytic anemia invariably entails thorough review by a hematologist, especially when splenomegaly is present because lymphoproliferative disorders are a differential diagnosis. We recommend testing patients with hemolytic anemia residing in regions endemic for *N*. *mikurensis* (i.e., large parts of Europe and northern Asia) for this emerging zoonotic infection that can easily be treated and cured by oral antibiotics.
